# The tumor suppressor role and ceRNA network of miR-1294 in cancer

**DOI:** 10.32604/or.2022.027359

**Published:** 2023-03-01

**Authors:** YUNAN MAO, JINZE SHEN, LI FANG, FENG ZHU, SHIWEI DUAN

**Affiliations:** 1 Department of Clinical Medicine, School of Medicine, Zhejiang University City College, Hangzhou, 310000, China; 2 Sir Run Run Shaw Hospital, College of Medicine, Zhejiang University, Hangzhou, 310016, China

**Keywords:** miR-1294, Expression, Cancer, ceRNA, Signaling pathway, Prognosis

## Abstract

miRNAs are endogenous small RNAs that are important regulators of gene expression. miR-1294 was found to be significantly down-regulated in 15 cancers and regulated by 21 upstream regulators. miR-1294 affects the proliferation, migration, invasion, and apoptosis of cancer cells. The target genes of miR-1294 are involved in the PI3K/AKT/mTOR, RAS, and JAK/STAT signaling pathways. Six target genes of miR-1294 are the targets of a variety of drugs. Low expression of miR-1294 is associated with resistance to cisplatin and TMZ and a poorer prognosis in patients with ESCC, GC, EOC, PDAC, or NSCLC. Therefore, this work outlines the molecular mechanisms and provides a basis for the clinical significance of the tumor suppressor miR-1294 in cancer.

## Introduction

As endogenous small RNAs, microRNAs (miRNAs) bind to the 3′ UTRs of their target messenger RNAs (mRNAs) to inhibit their expression, thereby affecting the development, differentiation, and progression of diseases [1,2]. Competitive endogenous RNAs (ceRNAs) such as long non-coding RNAs (lncRNAs) and circular RNAs (circRNAs) can compete with miRNAs [3], and thus regulate the expression of miRNAs and their targeted inhibition of protein-coding genes [4].

There are at least 19 target genes of miR-1294. The regulation of miR-1294 by ceRNA in various cancers can affect the expression of downstream target genes and various cellular behaviors of cancer cells. The downstream genes of miR-1294 are involved in the regulation of the phosphatidylinositol 3-kinase (PI3K)/AKT/mechanistic target of rapamycin kinase (mTOR), RAS, and Janus kinase (JAK)/signal transducer and activator of transcription (STAT) signaling pathways. Six target genes of miR-1294 are the targets of a variety of known drugs. This work provides a comprehensive summary of miR-1294, which provides potential directions for future research.

### Dysregulated miR-1294 in cancer

Previous studies have shown that miR-1294 is downregulated in 15 cancers, suggesting that elevated expression of miR-1294 may have anticancer potential. CeRNAs can compete with miRNAs to regulate the expression of protein-coding genes at the post-transcriptional level [5,6]. The ceRNAs of miR-1294 are highly expressed in 11 tumors, and by inhibiting the expression of miR-1294, they promote the occurrence and development of cancer (Tables 1 and 2). These ceRNAs are 3 lncRNAs and 16 circRNAs, including lncRNA TUG1 [7] in esophageal cancer (EC), KRT16P2 [8] in laryngeal squamous cell carcinoma (LSCC), and NEAT1 [9] in gastric cancer (GC); circRNAs include circ_0023984 in esophageal squamous cancer (ESCC) [10], circ_0005198 [11] and circ_0000936 [12] in glioma (GRAMD1A), and circAMOTL1 in oral squamous cell carcinoma (OSCC) [13], circ_0000885 [14] and circOMA1 [15] in osteosarcoma (OS), circ_0030235 [16] and circEYA3 [17] in pancreatic ductal adenocarcinoma (PDAC), circ_0004370 [18] in EC, circ_0000854 [19], circPRKCI [20], circCAMSAP1 [21], circUBAP2 [22], and circ_0000854 [23] in hepatocellular carcinoma (HCC), circPLK1 in malignant pleural mesothelioma (MPM) [24], circPLK1 [25] and circSHKBP1 [26] in non-small cell lung cancer (NSCLC), and circCDK17 [27], circ_0018289 [28] in cervical cancer (CC). Furthermore, miR-1294 was downregulated in ovarian cancer (OC) and clear cell renal cell carcinoma (ccRCC), thereby relaxing its repressive effects on insulin-like growth factor 1 receptor (IGF1R) and homeobox A6 (HOXA6).

**Table 1:** The aberrant expression of miR-1294 and its signaling axes in cancer

Type	Effect *in vitro*	Effect *in vivo*	Signaling axis
BC	Proliferation↓, invasion↓ and migration↓	Tumor volume↓ and tumor weight↓	–[32]
ESCC	Proliferation↓, invasion↓, migration↓ and cell cycle↓	–	circ_0023984/miR-1294/c-Myc [10,33]
GM	TMZ-resistance↓, proliferation↓, invasion↓, migration↓ and cell cycle↓	–	circ_0005198/miR-1294 [11]
			circ_0000936/miR-1294/TPX2 [12,34]
OSCC	Proliferation↓ and migration↓	–	circAMOTL1/miR-1294/ENO1|c-Myc|TRL4/6/8/9 [13,35]
OC	Cisplatin-resistance↓, proliferation↓, invasion↓ and migration↓	–	miR-1294/IGF1R [30]
GC	Proliferation↓, invasion↓, migration↓ and apoptosis↑	–	lncNEAT1/miR-1294/FOXK1|AKT1 [9,36]
OS	Proliferation↓, invasion↓, migration↓ and apoptosis↑	–	circOMA1/miR-1294/c-Myc [15]
			circ_0000885/miR-1294/FGFR1 [14]
			miR-1294/PKM2|HOXA9 [37,38]
PDAC	Proliferation↓, invasion↓, migration↓ and apoptosis↑	–	circ_0030235|circEYA3/miR-1294/c-Myc [16,17]
EC	Proliferation↓, invasion↓, migration↓ and apoptosis↑	-	lncTUG1/miR-1294/PLK1 [7]
			circ_0004370/miR-1294/LASP1 [18]
ccRCC	Proliferation↓ and invasion↓	–	miR-1294/HOXA6 [29]
HCC	Proliferation↓, invasion↓, migration↓ and apoptosis↑	Tumor growth↓	circ_0000854/miR-1294/IRGQ [19]
			circCAMSAP1/miR-1294/GRAMDA1 [21]
			circPRKCI/miR-1294/FOXK1 [20]
			circUBAP2/miR-1294/c-Myc|TEAD1|PIM1 [22,39]
LSCC	Proliferation↓, invasion↓ and migration↓	–	lncKRT16P2/miR-1294/EGFR [8]
MPM	Proliferation↓, invasion↓, migration↓ and stemness↓	Tumor growth↓	circPLK1/miR-1294/HMGA1 [24]
NSCLC	Proliferation↓, invasion↓, migration↓, apoptosis↑ and stemness↓	–	circSHKBP1/miR-1294/PKM2 [26]
			circPLK1/miR-1294/HMGA1 [25]
CC	Proliferation↓, invasion↓, migration↓ and apoptosis↑	Tumor volume↓ and tumor weight↓	circCDK17/miR-1294/YWHAZ [27]
			circ_0018289/miR-1294/ICMT [28]

Note: “↓” means that the biological behavior is inhibited, “↑” means that the biological behavior is promoted. Please check the full names of the abbreviations in the list of abbreviations. Downregulation of miR-1294 plays an important role in the ceRNA regulatory networks by relaxing the repression of target genes.

**Table 2:** The tested samples with aberrant expression of miR-1294

Type	miR-1294 expression	Level	Sample
**BC**	Lower in BC	Tissues and cells	30 BC tissues and matched normal tissues; BC cell lines (T47D, MDA-MB-468, BT474 and MCF-7) and the normal mammary epithelial cell line MCF-10A [32]
**ESCC**	Lower in ESCC	Tissues	ESCC tissues and matched normal tissues; ESCC cell lines (KYSE150, TE-1, and EC109) and an immortalized human esophageal epithelial cell line (Het-1A) [10,33]
**GM**	Lower in GM	Tissues and cells	normal human brain tissues and glioma specimens; normal human astrocytes (NHAs) and human glioma cell lines (U87, U251, LN229, and A172) [11,12,34]
**OSCC**	Lower in OSCC	Tissues and cells	24 OSCC tissues samples and matched adjacent normal tissues, 6 OLP and 6 OSCC tissues; primary gingival keratinocytes and OSCC cell lines (HSC2, HSC4, SAS, and KON) [13,35]
**OC**	Lower in OC	Tissues and cells	paired normal and OC cancer tissues; human OC cells SKOV3 [30]
**GC**	Lower in GC	Tissues and cells	172 GC tissues and adjacent normal tissues; human GC cell lines (SGC-7901, NCI-N87, HGC-27, MGC-803, and AGS) and normal gastric epithelial cell line GES-1 [9,36,40]
**OS**	Lower in OS	Tissues and cells	30 paired OS specimens and adjacent normal tissues; normal human osteoblastic cell line hFOB 1.19, human mesenchymal stem cells (hMSC1 and hMSC2) and OS cell lines (Saos-2, MG63, U2OS, HOS, and 143B) [14,38]
**PDAC**	Lower in PDAC	Tissues and cells	166 PDAC and matched non-cancerous tissues; PDAC cells (AsPC-1, BxPC-3, Capan-1, Capan-2, PANC1, SW1990, MiaPaCa-2, and CFPAC-1) and the normal cell line (HPDE) [16,17]
**EC**	Lower in EC	Tissues and cells	55 tumor tissues and adjacent normal tissues; esophageal cancer cell lines (ECA109, TE1, and KYSE-150) and human normal esophageal epithelial cells Het-1A [7,18]
**ccRCC**	Lower in ccRCC	Cells	human ccRCC cell lines Caki-1, Caki-2 and normal human renal tubular epithelial cell HK-2 [29]
**HCC**	Lower in HCC	Tissues and cells	125 HCC tissue samples and 40 para-cancerous normal tissues; HCC cell lines (HepG2, Hep3B, Huh-7, SMMC-7721, MHCC-97H, MHCC97L, and HCCLM3) and normal liver cell Lo-2 [19,21,22]
**LSCC**	Lower in LSCC	Tissues and cells	15 cases of LSCC tissues and 20 cases of adjacent normal tissues; human LSCC cell lines (TU212 and TU686) [8]
**MPM**	Lower in MPM	Tissues and cells	60 MPM tissues and 28 adjacent normal tissues; human MPM cell lines (MSTO-211H, H2373, H28, and H2052) and human normal mesothelial cell line LP-9 [24]
**NSCLC**	Lower in NSCLC	Tissues and cells	150 paired NSCLC tumors and their adjacent normal tissues; NSCLC cell lines (CALU3, PC9, H1650, CALU6, A549, H1229, and H1975) and human bronchial epithelial cell line HBE1 [25,26]
**CC**	Lower in CC	Tissues and cells	CC tissues and paired normal tissues; human CC cell lines (C-33A and HeLa) and human cervical epithelial cell line (Ect1/E6E7 and HcerEpic) [27,28]

Note: miR-1294 is lowly expressed in BC, ESCC, GM, OSCC, OC, GC, OS, PDAC, EC, ccRCC, HCC, LSCC, MPM, NSCLC, and CC. Please check the full names of the abbreviations in the list of abbreviations.

Notably, ceRNAs of miR-1294 have not been found in Breast cancer (BC), ccRCC, OC, and low expression of miR-1294 can relax the repression of HOXA6 [29], IGF1R [30], thereby promoting cancer risk. In addition, low expression of circLDLR in ovarian fluid significantly upregulated the expression of miR-1294, which was associated with the risk of polycystic ovary syndrome (PCOS) [31].

### Pan-cancer analysis of miR-1294

**Figure 1:**
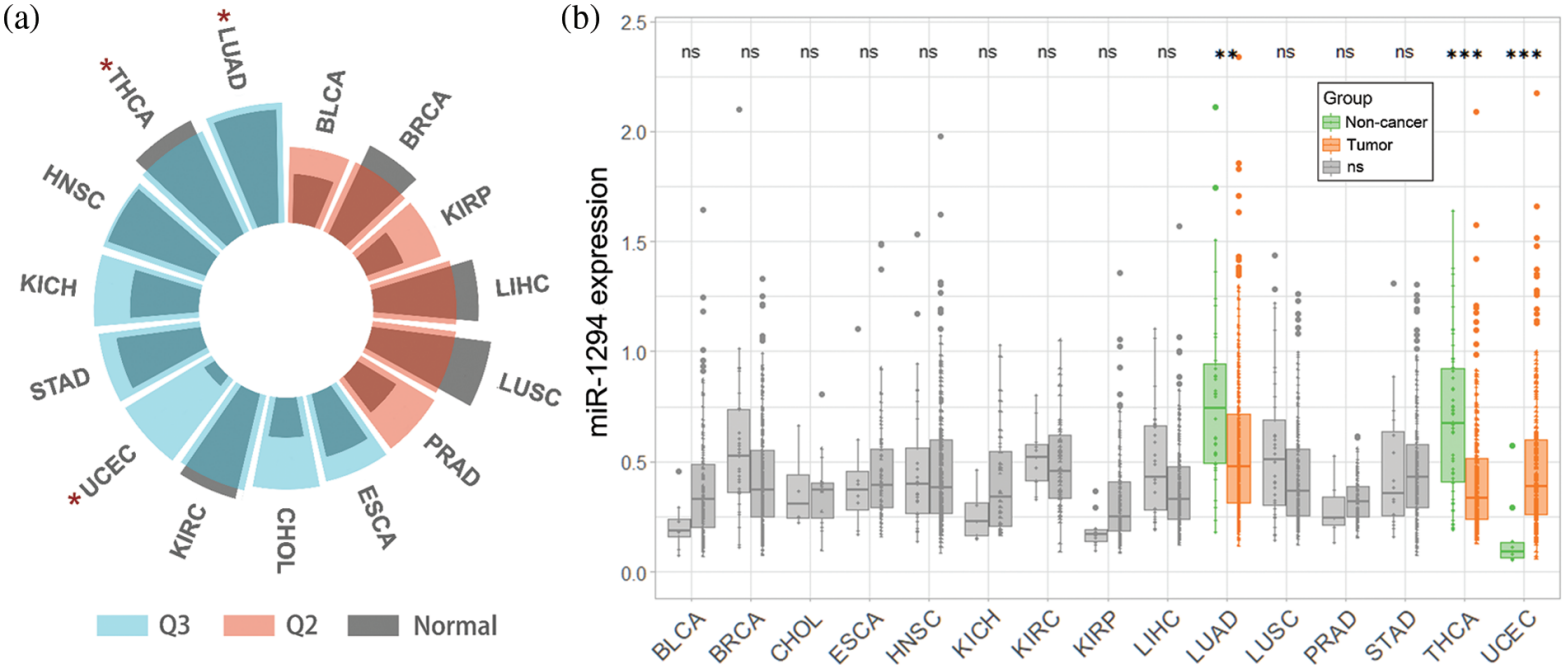
Pan-cancer analysis of miR-1294 using TCGA database. Please check the full names of the abbreviations in the list of abbreviations. a: * means there is a significant difference (*p* < 0.05) in the expression of miR-1294 between tumor and non-tumor samples. b: miR-1294 expression was Log2(RPM+1) transformed. *** means *p* < 0.001; ** means *p* < 0.01; * means *p* < 0.05; ns means no significant difference.

We downloaded the TCGA (pan-cancer) dataset from the UCSC Xena database (https://xenabrowser.net/). After removing cancer species without control samples, we performed a log2(x+1) transformation of the extracted miR-1294 expression data (RPM) in the samples, and we finally obtained miR-1294 expression data for 15 cancer types. In addition, we calculated the median expression of all miRNAs in each of the 15 cancers and calculated the quantile ranking of miR-1294 among all non-zero-expressed miRNAs. As shown in Fig. 1a, miR-1294 was highly expressed in 9 tumors including lung adenocarcinoma (LUAD), thyroid carcinoma (THCA), head and neck squamous cell carcinoma (HNSC), kidney chromophobe (KICH), stomach adenocarcinoma (STAD), uterine *corpus* endometrial carcinoma (UCEC), kidney renal clear cell carcinoma (KIRC), cholangiocarcinoma (CHOL), and esophageal carcinoma (ESCA) (0.5–0.75 quantile, Q3). miR-1294 was moderately expressed in 6 tumors (bladder urothelial carcinoma (BLCA), breast invasive carcinoma (BRCA), kidney renal papillary cell carcinoma (KIRP), liver hepatocellular carcinoma (LIHC), lung squamous cell carcinoma (LUSC), and prostate adenocarcinoma (PRAD)) (0.25–0.5 quantile, Q2). Finally, we calculated the difference in miR-1294 expression between normal and tumor samples of 15 cancers (unpaired Wilcoxon test).

Pan-cancer analysis showed that miR-1294 was downregulated in TCGA-LUAD and TCGA-THCA (Figs. 1a and 1b), which further validated the anticancer effect of miR-1294. Notably, miR-1294 was upregulated in TCGA-UCEC. Due to the small number of noncancerous samples involved (n = 10), the cancer-promoting effect of miR-1294 in TCGA-UCEC needs to be treated with caution.

Studies have shown that the expression of miR-1294 is significantly down-regulated in 7 cancers including BC, ESCC, EC, GC, ccRCC, HCC, and NSCLC. However, there was no significant association of miR-1294 expression with cancer risk among the corresponding TCGA cancer types (BRCA, ESCA, STAD, KIRC, LIHC, and LUSC) (Table 3).

**Table 3:** Comparison of miR-1294 and cancer risk associations between TCGA data and existing miR-1294 studies

Cancer type	Number of samples	miR-1294 expression in TCGA^#^	miR-1294 expression in the present studies*
BLCA	T = 139, N = 9	ns, Q2	–
BRCA	T = 267, N = 33	ns, Q2	Lower in BC tissues and cells (T47D, MDA-MB-468, BT474, and MCF-7) [32]
LIHC	T = 151, N = 32	ns, Q2	Lower in HCC cells (MCC-7721 and MHCC-97H) [21]
LUSC	T = 162, N = 41	ns, Q2	lower in NSCLC cells (CALU3, CALU6, A549, H1229, and H1975) [25]
PRAD	T = 84, N = 6	ns, Q2	–
KIRP	T = 110, N = 10	ns, Q2	–
CHOL	T = 15, N = 4	ns, Q3	–
ESCA	T = 80, N = 8	ns, Q3	Lower in ESCC tissues [33]; lower in EC cells (Eca-109 and KYSE-150) [18]
HNSC	T = 239, N = 29	ns, Q3	–
KICH	T = 36, N = 9	ns, Q3	–
KIRC	T = 62, N = 11	ns, Q3	Lower in ccRCC cells (Caki-1 and Caki-2) [29]
LUAD	T = 314, N = 38	Downregulation, Q3	–
STAD	T = 186, N = 16	ns, Q3	Lower in GC tissues and cells (NCI-N87 and AGS) [9]
THCA	T = 282, N = 52	Downregulation, Q3	–
UCEC	T = 211, N = 10	Upregulation, Q3	–

Note: #: Q2 and Q3 stand for 0.25–0.50 and 0.50–0.75 quantile expression. T and N denote tumor and normal tissues; ns means no significant difference; Please check the full names of the abbreviations in the list of abbreviations. *: Other miR-1294-related cancers (GM, ESCC, OC, OS, PDAC, LSCC, MPM, and CC) lack expression data in their TCGA counterparts and are therefore not shown.

These inconsistencies may be due to the following reasons. First, miR-1294-related studies are mostly based on cell lines with controlled heterogeneity. However, the heterogeneity of the cancerous and paracancerous tissues in TCGA was high. The proportion of cancer cells also varied among TCGA cancer tissue samples. Second, the expression level of miR-1294 was lower in various cancer types of TCGA (Q2-Q3). The expression of miR-1294 in TCGA was detected by RNA-seq technology. However, the existing miR-1294-related research generally uses qRT-PCR technology to amplify the target gene, and this method can detect very low expression of miR-1294. In addition, cell line-based studies involve more target RNA content and are more suitable for studying miR-1294, which is less expressed. Third, there may be highly expressed tissue-specific regulatory factors or ceRNAs, which significantly inhibit the expression level of miR-1294. And this affects the differential analysis of miR-1294 expression between cancerous and paracancerous tissues in TCGA. Taken together, the differences in the association results between miR-1294 expression and cancer risk may be related to different cancer tissue samples, gene expression detection methods, differences in sample numbers, and the presence of tissue-specific regulators such as ceRNAs. The anticancer effect of miR-1294 in more samples needs to be further verified in the future.

### Molecular mechanisms of miR-1294 affecting cancer cell behaviors

**Figure 2:**
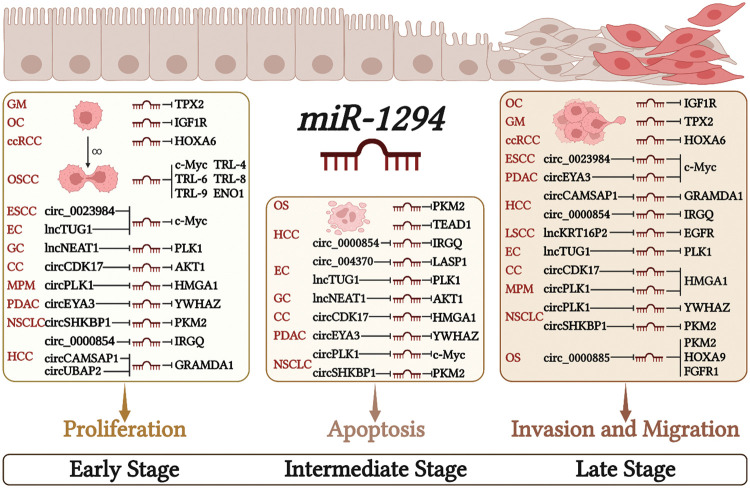
Molecular mechanisms by which miR-1294 affects cancer cell behaviors. Downregulation of miR-1294 promotes cell proliferation, invasion, and migration, and inhibits cancer cell apoptosis by regulating the expression of various target genes. Please check the full names of the abbreviations in the list of abbreviation.

The low expression of miR-1294 in cancer cells can relieve its inhibitory effect on downstream protein-coding genes, and then regulate the proliferation, apoptosis, invasion, and migration of cancer cells, and finally lead to the occurrence and development of cancer (Fig. 2).

Cell proliferation is an essential component of cell growth and differentiation [41]. Low expression of miR-1294 can up-regulate the expression of downstream protein-coding genes microtubule nucleation factor (TPX2) [34], IGF1R [30], MYC proto-oncogene, bHLH transcription factor (c-Myc) [33,35] and TRL4, TRL6, TRL8, TRL9 [35], enolase 1 (ENO1) [13], thereby promoting the proliferation of various tumor cells. In ESCC, PDAC, EC, GC, MPM, CC, NSCLC, and HCC, The highly expressed ceRNAs increase the expression of downstream protein-coding genes by inhibiting miR-1294, thereby promoting the proliferation of cancer cells. These ceRNA/miRNA/PCG signaling axes include circ_0023984/miR-1294/c-Myc in ESCC [10], circEYA3/miR-1294/c-Myc in PDAC [17], lncTUG1/miR-1294/PLK1 in EC [7], lncNEAT1/miR-1294/AKT serine/threonine kinase 1 (AKT1) in GC [9], circPLK1/miR-1294/high mobility group AT-hook 1 (HMGA1) in MPM [24], circCDK17/miR-1294/tyrosine 3-monooxygenase/tryptophan 5-monooxygenase activation protein zeta (YWHAZ) in CC [27], circSHKBP1/miR-1294/pyruvate kinase M2 (PKM2) in NSCLC [26], circCAMSAP1| CircUBAP2/miR-1294/GRAM domain containing 1A (GRAMD1) [21,22] and circ_0000854/miR-1294/immunity related GTPase Q (IRGQ) [23] in HCC.

Apoptosis is a form of programmed cell death that removes damaged cells in an orderly and efficient manner. Dysregulation of apoptosis machinery is a hallmark of cancer [42]. Low expression of miR-1294 inhibited cancer cell apoptosis (Fig. 2). In OS and HCC, under-expressed miR-1294 inhibits cancer cell apoptosis by upregulating pyruvate kinase M2 (PKM2) in OS [37] and TEA domain transcription factor 1 (TEAD1) and pim-1 proto-oncogene in HCC [39]. These ceRNA/miRNA/PCG signaling axes that inhibit cancer cell apoptosis include lncTUG1/miR-1294/PLK1 [7] and circ_0004370/miR-1294/LIM and SH3 protein 1 (LASP1) [18] in EC, lncNEAT1/miR-1294/AKT1 in GC [9], circPLK1/miR-1294/HMGA1 [25] and circSHKBP1/miR-1294/PKM2 [26] in NSCLC, circCDK17/miR-1294/YWHAZ in CC [27], and CircEYA3/miR-1294/c-Myc in PDAC [17].

Metastasis of cancer cells is a major cause of cancer death, and its initial steps are cancer cell migration and invasion into surrounding tissues and vasculature [43]. miR-1294 is closely associated with cell migration and invasion in cancer (Fig. 2). The low expression of miR-1294 can up-regulate the downstream target genes c-Myc [33], TPX2 [34], IGF1R [30], and HOXA6 [29] to promote the invasion and migration of ESCC, GM, OC, ccRCC tumor cells. These ceRNA/miRNA/PCG signaling axes that can promote tumor cell invasion and migration include circEYA3/miR-1294/c-Myc in PDAC [17], circ_0023984/miR-1294/c-Myc in ESCC [10], circCAMSAP1/miR-1294/GRAMD1 [21] and circ_0000854/miR-1294/IRGQ [23] in HCC, lncKRT16P2/miR- 1294/epidermal factor receptor (EGFR) in LSCC [8], lncTUG1/miR-1294/PLK1 in EC [7], circCDK17/miR-1294/YWHAZ in CC [27], circPLK1/miR-1294/HMGA1 in NSCLC [25] and MPM [24], and circSHKBP1/miR-1294/PKM2 in NSCLC [26].

### miR-1294-related signaling pathways

**Figure 3:**
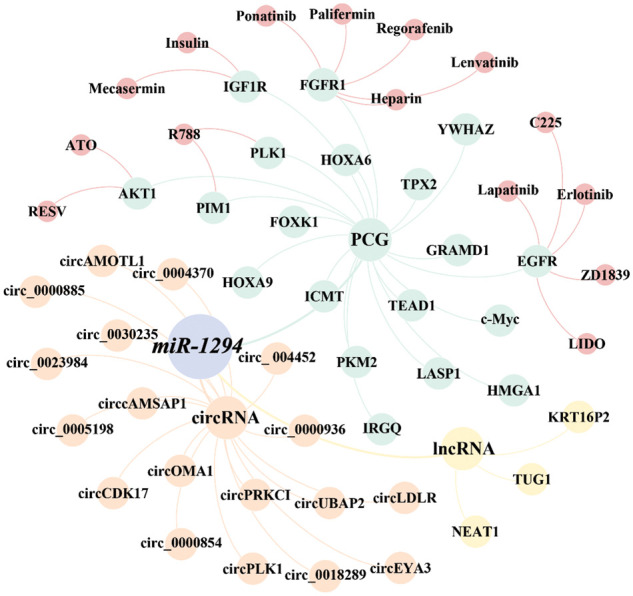
The ceRNA network and the druggable PCGs of miR-1294. The ceRNA network of miR-1294 includes 3 lncRNAs, 18 circRNAs, and 18 downstream PCGs, of which 6 PCGs have targeted drugs.

**Figure 4:**
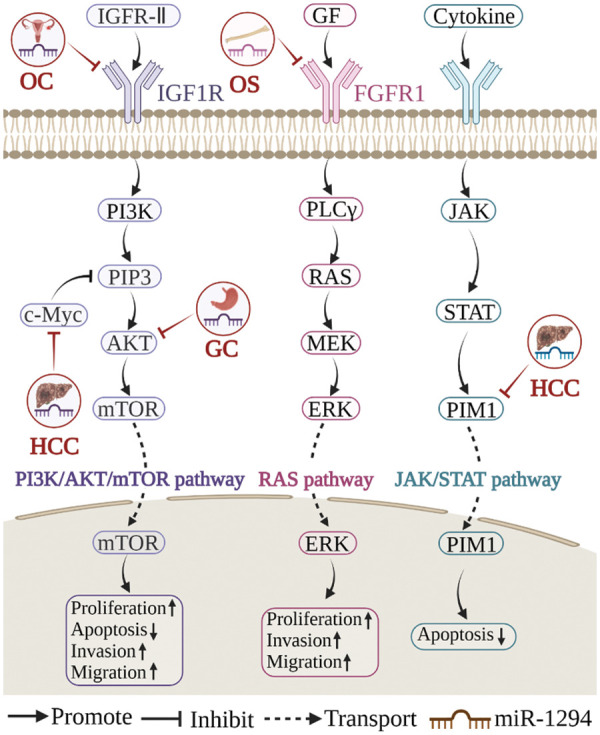
Three signaling pathways related to miR-1294. miR-1294 participates in three signaling pathways of PIK3/AKT/mTOR, RAS, and JAK/STAT to regulate cell biological processes.

miR-1294 inhibits the expression of at least 18 target genes (Fig. 3). Among them, five target genes (c-Myc, IGF1R, AKT, fibroblast growth factor 1 (FGFR1), and pim-1 proto-oncogene, serine/threonine kinase (PIM1)) are involved in the regulation of the PI3K/AKT/mTOR, RAS, JAK/STAT signaling pathways (Fig. 4), thereby affecting the proliferation, apoptosis, invasion, and progression of cancer cells.

### miR-1294 and PI3K/AKT/mTOR signaling pathway

The PI3K/AKT/mTOR signaling pathway is a master regulator of cancer [44], which is frequently activated in various cancers and is considered a promising therapeutic target [45]. In cisplatin-resistant tissues and cell lines (SKOV3/DDP) of OC, low expression of miR-1294 can increase the expression level of IGF1R, thereby mediating the activation of the PI3K/AKT/mTOR signaling pathway and promoting the proliferation, migration, and invasion of OC cells [30]. In HCC, CircUBAP2 acts as a sponge for miR-1294, upregulates c-Myc expression, and inhibits PI3P, thereby inhibiting the PI3K/AKT/mTOR signaling pathway and promoting tumorigenesis [22]. In GC, LncRNA NEAT1 increased the expression level of AKT1 by sponging miR-1294, mediated the activation of the PI3K/AKT/mTOR signaling pathway, promoted the proliferation and migration of GC cells, and inhibited apoptosis [9].

### miR-1294 and RAS signaling pathway

The RAS signaling pathway can control cell growth, survival, and differentiation by integrating extracellular signals. Aberrant activation of the RAS pathway is a highly prevalent major oncogenic event [46]. Circ_0000885, which is highly expressed in OS, can restore the expression level of FGFR1 by targeting miR-1294, thereby mediating the activation of the RAS signaling pathway and promoting the progression of OS [14].

### miR-1294 and JAK/STAT signaling pathway

The JAK/STAT signaling pathway is a mechanism by which extracellular factors regulate gene expression and is involved in many key biological processes such as cell proliferation, differentiation, apoptosis, and immune regulation [47]. Arsenic trioxide (ATO) is the most toxic compound in traditional Chinese medicine and has been shown to effectively inhibit cancer cell processes. In HCC, ATO induced the upregulation of miR-1294, decreased the expression level of PIM1, and inhibited the JAK/STAT signaling pathway, thereby promoting the apoptosis of HCC cells [39].

### The clinical significance of miR-1294

As shown in Table 4, the abnormal expression of miR-1294 in cancer is not only correlated with tumor prognostic indicators but also closely related to the clinicopathological phenotype of cancer patients. Cancer therapeutic drugs can target cancer by targeting the downstream genes of miR-1294. In addition, studies have also shown that low expression of miR-1294 is also associated with resistance to cisplatin and TMZ.

**Table 4:** Prognostic values of miR-1294 in cancer

Types	miR-1294 expression	Sample size	Clinicopathological characteristics	Prognostic value	Ref.
**ESCC**	Downregulation	79	Larger tumor size, positive lymphatic invasion, and positive venous invasion	Shorter 5-year overall survival	[33]
	Downregulation	44	Larger tumor diameter, lymph node metastasis	–	[10]
**GC**	Downregulation	60	–	Shorter overall survival	[36]
	Downregulation	82	Larger tumor size, distant metastasis, and lymph node metastasis	Shorter overall survival and disease-free survival	[40]
**EOC**	Downregulation	69	Advanced FIGO stage and lymph node metastasis	Shorter overall survival	[48]
**PDAC**	Downregulation	104	Advanced TNM stage	Shorter overall survival	[17]
	Downregulation	62	Higher tumor stage and positive lymph node invasion	Shorter overall survival	[16]
**NSCLC**	Downregulation	50	Advanced TNM and distant metastasis	Shorter overall survival	[25]

Note: In ESCC, GC, EOC, PDAC, and NSCLC, the low expression of miR-1294 is associated with clinicopathological characteristics and prognosis of tumor patients. Please check the full names of the abbreviations in the list of abbreviations.

### The diagnostic and prognostic value of miR-1294

As shown in Table 4, miR-1294 was down-regulated in most cancers, and its abnormal expression correlated with prognostic indicators of tumors. In ESCC, the overall survival rate of the miR-1294-low-expression group was significantly lower than that of the miR-1294-high-expression group [33]. In GC, patients with low miR-1294 expression had significantly shorter overall survival [36,40] and disease-free survival (DFS) [40] than patients with high expression of miR-1294 [36]. In EOC, the overall survival rate of the miR-1294-low-expression group was lower compared with the miR-1294-high-expression group [48]. The expressions of CircEYA3 and Circ_0030235 were significantly up-regulated in PDAC tissues compared with adjacent normal tissues. Survival analysis showed that the overall survival rate of PDAC patients with high expression of CircEYA3 and Circ_0030235 group was lower [17,16], thus indicating that in PDAC, the group with low expression of miR-1294 had lower overall survival rate. In NSCLC, the overall survival rate was lower in the miR-1294-low-expression group compared with the miR-1294-high-expression group [25].

### The relationship between miR-1294 and clinicopathological characteristics

As shown in  Table 4, the expression level of miR-1294 was closely related to the clinicopathological phenotype of cancer patients. In ESCC, low expression of miR-1294 was associated with larger tumors, positive lymphatic infiltration, lymph node metastasis, and positive venous infiltration [33,10]. In GC, low expression of miR-1294 was associated with larger tumors, lymph node metastasis, and distant metastasis [40]. In EOC, low expression of miR-1294 was associated with advanced FIGO stage and lymph node metastasis [48]. In PDAC, low expression of miR-1294 was associated with advanced TNM stage [17], higher tumor stage, and positive lymph node invasion [16]. In NSCLC, low expression of miR-1294 was associated with advanced TNM staging and distant metastasis in NSCLC patients [25].

### miR-1294 and cancer therapy

As shown in Fig. 3, we found that currently listed drugs can target 6 downstream genes of miR-1294 via the CADDIE website (https://exbio.wzw.tum.de/caddie/drug-lookup) [49]. These drugs are Palifermin, Heparin, Regorafenib, Ponatinib, and Lenvatinib targeting FGFR1, R788 (Fostamatinib) targeting PLK1 and PIM1, C225 (Cetuximab), LIDO (Lidocaine), (ZD1839) Gefitinib targeting EGFR, Erlotinib, and Lapatinib; Insulin and Mecasermin targeting IGF1R, and ATO and RESV (Resveratrol) targeting AKT1. In the future, it is necessary to confirm whether miR-1294 interacts with these drugs (Table 5).

**Table 5:** Binding sites of miR-1294 on ceRNAs and target genes

Type	ceRNA	Binding site of ceRNAs (5′-3′)	Binding site of miR-1294 (3′-5′)	Target gene	Binding site of target gene (5′-3′)	Binding site of miR-1294 (3′-5′)	Ref.
**ESCC**	–	–	–	c-Myc	AAUGCaAC CUCAC	UUACGguUG GAGUG	[33]
	circ_0023984	CCUCAC	GGAGUG				[10]
**GM**	circ_0005198	CCUCAC	GGAGUG	–	–	–	[11]
	circ_0000936	ACCUCAC	UGGAGUG				[12]
	–	–	–	TPX2	AGCCUC	GUUGGAG	[34]
**OSCC**	circAMOTL1	CAcgGaaAACCUCAC	GUuaCggUUGG AGUG	ENO1	UuCUcGCCU CAC	AcGGuUGGA GUG	[13]
	–	–	–	c-Myc	ACCUCAC	UGGAGUG	[35]
				TLR4	ACCUCAC	UGGAGUG	
				TLR6	CCUCACA	GGAGUGU	
				TLR8	ACCUCACC	UGGAGUGU	
				TLR9	ACCUCAC	UGGAGUG	
**OC**	–	–	–	IGF1R	CCUCAC	GGAGUG	[30]
**GC**	lncRNA NEAT1	AAUucuuACCUCACU	UUAcgguUGG AGUG	AKT1	CCUCAC	GGAGUG	[9]
	–	–	–	FOXK1	CCUCAC	GGAGUG	[36]
**OS**	circ_0000885	CCAACCUCAC	GGUUGGAGUG	FGFR1	ACCUCAC	UGGAGUG	[14]
	–	–	–	HOXA9	ACCUCAC	UGGAGUG	[38]
				PKM2	AAgAAgaUCAacGCCUCAC	UUgUUacGGUUGGAGUG	[37]
	circOMA1	ACAuUAGcaUCcACCUCAC	UGUuGUUacGG uUGGAGUG	c-Myc	AAUGCaACC UCACA	UUACGguGG AGUGU	[15]
**PDAC**	circ_0030235	CCUCAC	GGAGUG	–	–	–	[16]
		CUUCUC	GAAGAG				
	circEYA3	CAAUGauuau ACCUCACA	GUUACgguUGGAGUGU	c-Myc	AAUGCaACC UCACA	UUACGgUGG AGUGU	[17]
**EC**	circ0004370	UGGAGUG	ACCUCAC	LASP1	CCUCAC	GGAGUG	[18]
	lncRNA TUG1	AACAAcCCAc ACCUCAC	UUGUUacGGUUGGAGUG	PLK1	AcuggUGCCcuC CUCAC	UuguuACGGuuG GAGUG	[7]
**ccRCC**	–	–	–	HOXA6	ACCUCAC	UGGAGUG	[29]
**HCC**	–	–	–	TEAD1	AACCUCAC	UUGGAGUG	[39]
				PIM1	ACCUCAC	UGGAGUG	[39]
	circUBAP2	CCUCAC	GGAGUA	c-Myc	CCUCAC	GGAGUA	[22]
	circPRKCI	UCGACCUCAC	GGUUGGAGUG	FOXK1	AGCCUCAC	UUGGAGAG	[20]
	circCAMSAP1	CgAggATGCCAtggT	GuUguUACGGUu ggA	GRAMDA1	CCAACC	GGUUGG	[21]
	circ_0000854	ACCUCAC	UGGAGUG	IRGQ	ACAAauggcaucuACCUCAC	UGUUguuacgguU GGAGUG	[23]
**LSCC**	lncRNA KRT16P2	ACAATGCCAggCTggCA	TGTTACGGTtg GAgtGT	EGFR	CCUCAC	GGAGTG	[8]
**MPM**	circPLK1	ACCUCAC	UGGAGUG	HMGA1	CCUCAC	GGAGUG	[24]
**NSCLC**	circPLK1	ACCUCAC	UGGAGUG	HMGA1	CCUCAC	GGAGUG	[25]
**CC**	circCDK17	CCUCACA	GGAGUGU	YWHAZ	ACCUCAC	UGGAGUG	[27]
	circ_0018289	CCUCAC	GGAGUG	ICMT	ACCUCAC	UGGAGUG	[28]

Note: Please check the full names of the abbreviations in the list of abbreviations. Unpaired sequences are lowercase.

### miR-1294 and drug resistance

**Figure 5:**
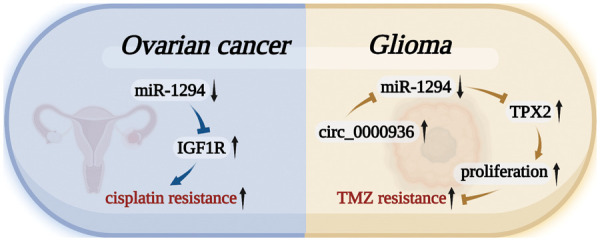
miR-1294 affects cellular drug resistance by inhibiting target genes. In OC tumor cells, the low expression of miR-1294 increased the resistance of OC cancer cells to cisplatin by upregulating the expression of the target gene IGF1R. In GM, the highly expressed Circ_0000936 can down-regulate the expression level of miR-1294 to up-regulate the expression of TPX2, promote the proliferation of GM cancer cells, and increase the resistance of GM cancer cells to TMZ.

miR-1294 was closely associated with cisplatin and TMZ resistance in cancer cells (Fig. 5). miR-1294 can affect the drug resistance of tumor cells by regulating targets, activating signaling pathways, or changing the normal behavior of molecules in two tumor cells.

Cisplatin is a well-known chemotherapy drug that has been used to treat a variety of human cancers [50]. The development of cisplatin chemoresistance can lead to the failure of cisplatin therapy [51]. In OC, miR-1294 was significantly decreased in tissues of cisplatin-resistant patients compared with cisplatin-sensitive patients. *In vitro*, miR-1294 also showed low expression in cisplatin-resistant cell lines (SKOV3/DDP) compared with OC SKOV3 cells. Low expression of miR-1294 can restore the expression level of the target gene IGF1R and activate the PI3K/AKT/mTOR signaling pathway, thereby upregulating the cisplatin resistance of OC cells [30].

Temozolomide is used as an oral alkylating agent in the treatment of glioblastoma multiforme (GBM) and astrocytoma [52]. miR-1294 expression was lower in high-grade gliomas than in low-grade gliomas. Low-expressed miR-1294 upregulates the expression of TPX2, which promotes the proliferation, migration, and invasion of GM cells, and reduces the chemosensitivity of GM cells to temozolomide [34]. Meanwhile, the expression of Circ_0000936 in temozolomide-resistant GM tissues was higher than that in temozolomide-sensitive GM tissues. The highly expressed Circ_0000936 can down-regulate the expression level of miR-1294, thereby increasing the resistance of GM cells to TMZ [12].

## Discussion

**Figure 6:**
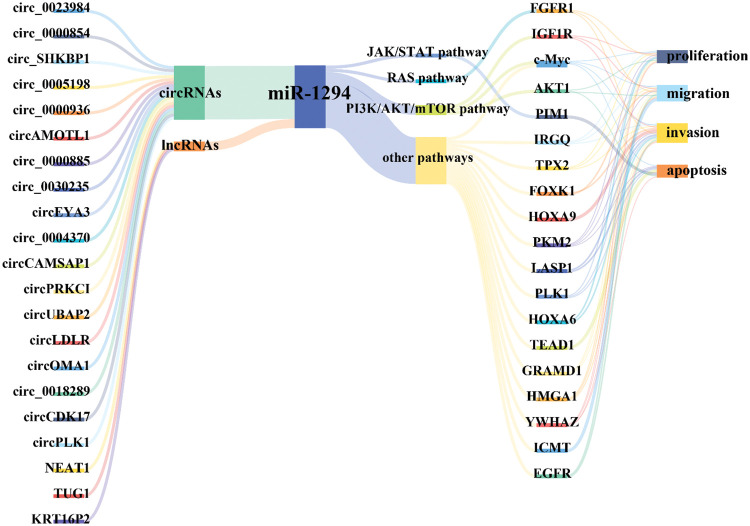
Molecular mechanism of miR-1294-centered ceRNA network. Under the regulation of multiple ceRNAs, inhibition of miR-1294 can relax the down-regulation of its target genes, thereby regulating the migration, proliferation, invasion, and apoptosis of cancer cells. Please check the full names of the abbreviations in the list of abbreviations.

Available evidence indicates that miR-1294 expression is downregulated in 15 tumors, including BC, ESCC, OC, ccRCC, GM, OSCC, GC, OS, PDAC, EC, HCC, LSCC, MPM, NSCLC, and CC. miR-1294 has 21 upstream ceRNAs (including 18 circRNAs and 3 lncRNAs) and 19 downstream target genes (Fig. 6). Low expression of miR-1294 can promote the proliferation, apoptosis, invasion, and migration of cancer cells, and can participate in the activation of PI3K/AKT/mTOR, RAS, JAK/STAT signaling pathways, and promote the development of cancer. Down-regulation of miR-1294 was associated with poorer prognosis in ESCC, GC, EOC, PDAC, and NSCLC. In addition, low expression of miR-1294 was also associated with resistance to cisplatin and TMZ.

Notably, the analysis of TCGA also found that miR-1294 was down-regulated in TCGA-LUAD and TCGA-THCA, while its expression was up-regulated in TCGA-UCEC. Furthermore, miR-1294 expression was upregulated in the noncancerous disease PCOS. The functional differences of miR-1294 may be related to mechanisms such as differences in samples, miRNA detection methods, differences in sample numbers, and the presence of tissue-specific regulators such as ceRNAs.

Low expression of miR-1294 in ovarian cancer and glioma is associated with TMZ and cisplatin resistance. Porous lyotropic liquid crystal nanoparticles are promising delivery vehicles for cancer therapy [19]. The use of targeted nanomedicine to deliver miR-1294 may have great potential for cancer therapy.

However, there are still many deficiencies in the current research on miR-1294. First, the number of current research samples is small, and relevant results need to be verified in larger samples and other populations. Secondly, some studies on the biological functions of miR-1294 are limited to *in vitro* cell experiments, and it is necessary to strengthen the verification of *in vivo* animal experiments in the future. Finally, the molecular mechanism of miR-1294 in disease is still not fully understood, and more in-depth research is needed in the future to provide a theoretical basis for miR-1294-targeted therapeutic regimens.

## Conclusion

As a tumor suppressor, the low expression of miR-1294 has an important molecular regulatory mechanism in cancer cell behavior and carcinogenesis. In addition, the overview of miR-1294 in cancer diagnosis, prognosis, and treatment is expected to provide potential clues and directions for miR-1294-related clinical research.

## Data Availability

All data generated or analyzed during this study are included in the article.

## Abbreviations

ATO: Arsenic trioxide
AKT1: AKT serine/threonine kinase 1
BC: Breast cancer
BLCA: Bladder urothelial carcinoma
BRCA: Breast invasive carcinoma
CC: Cervical cancer
ccRCC: Clear cell renal cell carcinoma
ceRNA: Competitive endogenous RNA
CHOL: Cholangiocarcinoma
CircRNA: Circular RNA
c-Myc: MYC proto-oncogene, bHLH transcription factor
DFS: Disease-free survival
EC: Esophageal cancer
EGFR: Epidermal factor receptor
ENO1: Enolase 1
ESCA: Esophageal carcinoma
ESCC: Esophageal squamous cell carcinoma
FGFR1: Fibroblast growth factor 1
FOXK1: Forkhead box K1
GBM: Glioblastoma multiforme
GC: Gastric cancer
GM: Glioma
GRAMD1A: GRAM domain containing 1A
HCC: Hepatocellular carcinoma
HMGA1: High mobility group AT-hook 1
HNSC: Head and neck squamous cell carcinoma
HOXA6: Homeobox A6
ICMT: Isoprenylcysteine carboxyl methyltransferase
IGF1R: Insulin-like growth factor 1 receptor
IRGQ: Immunity related GTPase Q
JAK: Janus kinase
KICH: Kidney chromophobe
KIRC: Kidney renal clear cell carcinoma
KIRP: Kidney renal papillary cell carcinoma
LASP1: LIM and SH3 protein 1
LIHC: Liver hepatocellular carcinoma
LncRNA: Long non-coding RNA
LSCC: Laryngeal squamous cell carcinoma
LUAD: Lung adenocarcinoma
LUSC: Lung squamous cell carcinoma
mRNA: Messenger RNA
miRNA: MicroRNA
MPM: Malignant pleural mesothelioma
mTOR: Mechanistic target of rapamycin kinase
NSCLC: Non-small cell lung cancer
OC: Ovarian cancer
OS: Osteosarcoma
OSCC: Oral squamous cell carcinoma
PCOS: Polycystic ovary syndrome
PDAC: Pancreatic ductal adenocarcinoma
PIM1: Pim-1 proto-oncogene, serine/threonine kinase
PI3K: Phosphatidylinositol 3-kinase, putative
PKM2: Pyruvate kinase M1/2
PRAD: Prostate adenocarcinoma
STAD: Stomach adenocarcinoma
STAT: Signal transducer and activator of transcription
TEAD1: TEA domain transcription factor 1
THCA: Thyroid carcinoma
TPX2: TPX2 microtubule nucleation factor
UCEC: Uterine *corpus* endometrial carcinoma
YWHAZ: Tyrosine 3-monooxygenase/tryptophan 5-monooxygenase activation protein zeta
